# Genes Induced by Panax Notoginseng in a Rodent Model of Ischemia-Reperfusion Injury

**DOI:** 10.1155/2020/8873261

**Published:** 2020-11-25

**Authors:** Lanqing Meng, Qing Huang, Xuebin Li, Ping Liang, Yueyong Li, Xiaohua Huang, Jingjie Zhao, Qiuping Chen, Rong Qiu, Lan Li, Chongdong Jian, Hongfei Yao, Jianmin Huang, Xionglin Tang, Zechen Wang, Zhongheng Wei, Jun Wu, Liuzhi Wei, Qiuju Wei, Qianli Tang, Lu Huang, Jihua Wei, Dinggui Lu, Qunqiang Luo, Kegong Xie, Yang Ouyang, Jian Chen, Genliang Li, Linxue Luo, Linbo He, Chenyi Zhuo, Anding Xu, Lingzhang Meng

**Affiliations:** ^1^Jinan University, Guangzhou City, Guangdong Province, China; ^2^Department of Neurology, Affiliated Hospital of Youjiang Medical University for Nationalities, Baise City, Guangxi Province, China; ^3^Graduate School of Youjiang Medical University for Nationalities, Baise City, Guangxi Province, China; ^4^Department of Neurology, The First People's Hospital of Yulin, Yulin City, Guangxi Province, China; ^5^Department of Inverventional Medicine, Affiliated Hospital of Youjiang Medical University for Nationalities, Baise City, Guangxi Province, China; ^6^Life Science and Clinical Research Center, Affiliated Hospital of Youjiang Medical University for Nationalities, Baise City, Guangxi Province, China; ^7^Department of Rehabilitation Medicine, Affiliated Hospital of Youjiang Medical University for Nationalities, Baise City, Guangxi Province, China; ^8^Center for Systemic Inflammation Research (CSIR), School of Preclinical Medicine, Youjiang Medical University for Nationalities, Baise City, Guangxi Province, China; ^9^Urinary Surgery Department, Affiliated Hospital of Youjiang Medical University for Nationalities, Baise City, Guangxi Province, China; ^10^College of Pharmacy, Youjiang Medical University for Nationalities, Baise City, Guangxi Province, China; ^11^Trauma Center, Affiliated Hospital of Youjiang Medical University for Nationalities, Baise City, Guangxi Province, China; ^12^Department of Biochemistry and Molecular Biology, Youjiang Medical University for Nationalities, Baise City, Guangxi Province, China; ^13^Department of Gynaecology and Obstetrics, Affiliated Hospital of Youjiang Medical University for Nationalities, Baise City, Guangxi Province, China; ^14^Shangsi People's Hospital, Fangchenggang City, Guangxi Province, China; ^15^Department of Hepatological Surgery, Affiliated Hospital of Youjiang Medical University for Nationalities, Baise City, Guangxi Province, China

## Abstract

Stroke is a cerebrovascular disease that results in decreased blood flow. Although Panax notoginseng (PN), a Chinese herbal medicine, has been proven to promote stroke recovery, its molecular mechanism remains unclear. In this study, middle cerebral artery occlusion (MCAO) was induced in rats with thrombi generated by thread and subsequently treated with PN. After that, staining with 2,3,5-triphenyltetrazolium chloride was employed to evaluate the infarcted area, and electron microscopy was used to assess ultrastructural changes of the neurovascular unit. RNA-Seq was performed to determine the differential expressed genes (DEGs) which were then verified by qPCR. In total, 817 DEGs were identified to be related to the therapeutic effect of PN on stroke recovery. Further analysis by Gene Oncology analysis and Kyoto Encyclopedia of Genes and Genomes revealed that most of these genes were involved in the biological function of nerves and blood vessels through the regulation of neuroactive live receptor interactions of PI3K-Akt, Rap1, cAMP, and cGMP-PKG signaling, which included in the 18 pathways identified in our research, of which, 9 were reported firstly that related to PN's neuroprotective effect. This research sheds light on the potential molecular mechanisms underlying the effects of PN on stroke recovery.

## 1. Introduction

Stroke is a major cause of death in the world, and it can also lead to long-term disability [1]. Ischemic stroke is due to cerebral artery occlusion, which interrupts the blood supply of the brain, resulting in hypoxia and a lack of nutrients, proceeded by a series of complex pathological changes. A common feature of ischemic stroke is cerebral ischemia and reperfusion injury (CIRI) which results in serious damage (Eltzschig and Eckle, 2011), including pathological processes such as excitatory glutamate toxicity, energy failure, free radical formation, oxidative stress, inflammatory response, Ca^2+^ overload, and apoptosis [2]. The concept of neurovascular unit (NVU) was first recognized in 2001, and alterations in the composition of the NVU have been shown to increase vulnerability to the damaging effects of ischemic stroke [3]. While the NVU has become an integral component in the study of biomarkers of ischemic stroke [4], effective neuroprotective drug targets in CIRI are yet to be determined.

*Panax notoginseng* is a precious Chinese herbal medicine, which is grown mainly in Wenshan Prefecture, Yunnan Province, China. It has been reported that at least twenty saponins were contained in this material, including notoginsenoside R1, ginsenoside Rb1, and ginsenoside Rg1 [5, 6]. It is anti-inflammatory and antioxidative, and it is able to regulate the balance of neurotransmitters and promoting the regeneration of nerves and blood vessels [7–9]. Our previous studies have shown that Panax notoginseng saponins (PNS), an extract of PN, have both anti-inflammatory and neuroprotective effects [10]. Previously, it has been reported that PN exerted neuroprotective effect in stroke through antioxidative and anti-inflammatory properties [11]; in combination with Angelica sinensis, it could also inhibit NF-*κΒ* signaling and DNA binding activity, downregulate NO, NLRP3 inflammasome formation, and influence microglial pyroptosis [12, 13]. PN may also influence the expression of Nogo-A, NgR, and p75, regulate NgR1/RhoA/Rock2 pathway, thus contribute to the recovery of nerve function in stroke [14, 15]. However, further studies need to be conducted to explore underlying mechanism(s) of PN's protective effects on NVU, especially in stroke.

In this study, we first evaluated the effect of PN on the ultrastructure of the NVU, and then used the RNA-sequencing methods to study the gene expression changes caused by PN on the brain ischemia-reperfusion injury rats. The differentially expressed genes (DEGs) and their signal pathways were also examined in an attempt to gain a better understanding of all the biochemical mechanisms involved in this process.

## 2. Materials and Methods

### 2.1. Animal and Experimental Design

Sprague-Dawley (SD) female rats (250 ± 30 g) were purchased from Changsha Tianqin Biotechnology Co., Ltd, (Hunan, China). Before the experiments, all rats were maintained under standard laboratory conditions (a 12 : 12 hours of day and night cycle, a relative humidity of 60 ± 5% at room temperature of 22 ± 2°C and free access to food and water). This research was approved by the Animal Experimental Ethics Committee of Youjiang Medical University for Nationalities.

The middle cerebral artery occlusion (MCAO) model has been described in detail [16]. Briefly, we exposed the right internal and external carotid arteries; then, we cut external carotid artery about 3 mm above the common carotid artery bifurcation. After a silk suture was tied around the external carotid stump, a nylon filament (diameter: 0.265 mm, rounded tip, dipped in heparin) was then inserted into the external carotid artery and gently advanced into the internal carotid artery, 17-19 mm from the carotid bifurcation until a detection of slight resistance. A nylon thread was then tied into the vascular lumen. After sterilization, suture incision and following 2 h of ischemia, the thrombus was pulled out for about 1 cm to complete reperfusion injury. Rats in the sham group underwent the same surgical procedures except for the nylon thread procedure. The ambient temperature was kept constant by maintaining the rectal temperature in the rats at 37 ± 1°C.

After being allowed to adapt to the environment for 7 days, at least 10 animals were randomly distributed in each group. The intervention group was administrated with PN (Sigma-Aldrich, #1291719) intragastrically at a dose of 100 mg·kg-1 every 12 hours. The dose in this study was chosen based on previous study [13] and our preliminary experiments. Basically, 3 groups were designed in our study: the Sham group, the MCAO group which treated with saline for 7 days, and the MCAO group which treated with PN for 7 days. Both saline and PN administration started 2 hours after surgery. All rats were fasted but allowed free access to water.

### 2.2. Evaluation of Neurological Defects

Neurobehavioral impairment of rats was assessed in a double-blind manner by two independent observers according to Longa's [16]. Detailed information of this evaluation method was summarized in the supplementary table (available here).

The rats with 1-3 points were considered as successful modeling for I/R injury and were scored again for validation 7 days after surgery.

After 7 days, 6 rats were selected from each group and anesthetized. The rats in this study were anesthetized by IP injection of Ketamine (80 mg/kg) and Xylazine (8 mg/kg); supplemental heat was provided to avoid hypothermia during anesthesia, according to IACUC guidelines [17, 18]. Brain tissue was separated rapidly, and the area of cerebral infarction was measured using 2,3,5-triphenyltetrazolium chloride (TTC) staining in order to show the impaired area of cerebral ischemia.

### 2.3. Ultrastructural Changes of NVU [19, 20]

7 days after the procedure, the cortex around the ischemic focus (1 mm × 1 mm × 1 mm) was immersed in 2.5% glutaraldehyde for 2-4 hours and washed 3 times with 0.1 M phosphate buffer (pH 7.4) for 15 minutes. The samples were fixed in osmic acid for 2 hours at room temperature, washed 3 times with 0.1 M phosphate buffer (pH 7.4) for 15 minutes, and fixed and stored at 4°C. Subsequently, samples were dehydrated in an alcohol gradient (50%-70%-80%-90%-95%-100%-100% ethanol-100% acetone-100% acetone) and embedded in EPON 812 epoxy resin. Ultrathin sections of 50 nm were stained with 3% saturated solution of uranyl acetate and 6% lead citrate staining solution. The ultrastructure of neurons, astrocytes, and endothelial cells was observed using a JEOL-1011 transmission electron microscope (JEOL, Japan) at 80 kV.

### 2.4. RNA Extraction, Library Construction, and Sequencing

According to the manufacturer's protocol, total RNA was extracted with Trizol kit (Invitrogen, USA). RNA quality was evaluated on the Agilent 2100 Bioanalyzer (Agilent Technologies, USA) and verified by RNase-free agarose gel electrophoresis. After extraction, oligo (dT) beads were used to enrich eukaryotic mRNA; then, Ribo-ZeroTM magnetic kit (Epicentre, USA) was used to remove rRNA.

The mRNA acquired above was treated with fragmentation buffer, then reverse transcribed into cDNA. Second-strand cDNA was synthesized with DNA polymerase I, RNase H, and dNTP, and then purified with a QiaQuick PCR extraction kit (Qiagen, Netherlands); end-repaired, poly(A) tails were added and ligated to Illumina sequencing adapters. The ligation products were separated and selected based on size with agarose gel electrophoresis, and PCR amplification and sequencing were performed with Illumina HiSeq2500.

### 2.5. Assembling and Processing Sequencing Raw Data

Clean readings were obtained by removing reads which contain adapters, those with more than 10% of unknown nucleotides (N) and low-quality reads containing more than 50% of low quality (*Q* value ≤ 20) bases. Using StringTie v1.3.1 [21, 22], transcripts were assembled from sequenced raw data processed by the HISAT2. 2.4 [23] method. For each transcription region, a fragment per kilobase of transcript per million mapped reads (FPKM) [24] value was calculated to quantify its expression abundance and variation, using the StringTie software.

### 2.6. Differentially Expressed Genes

RNAs differential expression analysis was performed by using DESeq2 [25] software between two different groups (and by edge R [26] between two samples). The genes with a false discovery rate (FDR) below 0.05 and an absolute fold-change ≥ 2 were considered to be DEGs. qPCR was performed in order to verify the expression of these screened DEGs between the different groups.

### 2.7. Validation of the RNA-Sequencing Results by qPCR

To verify the reliability of sequencing results, qPCR was used to verify gene expression in the same batches of MCAO and PN samples (*n* = 6), and *β*-actin was used as an internal reference. The 20 *μ*L reaction system contained 5 *μ*L of cDNA, 0.5 *μ*L of each primer, 0.5 *μ*L 2 x SYBR Green qPCR SuperMix (InVitrogen), and 4 *μ*L dH_2_O, and the reaction conditions were as follows: 50°C for 2 min, 95°C for 2 min, 95°C for 15 s, and 60°C for 32 s plate read for 40 cycles followed by melting curve analysis (60°C to 95°C). The 2^−*△△*Ct^ method was used to determine the relative amount of mRNA, and 3 measurements were made for each sample. Primers used for Cttn amplification are 5′-ATGTGGAAAGCTTCAGCAGGCC-3′ (forward) and 5′-TCACGGGCACT CCGGGACCCAA-3′ (reversed); primers used for Cxcl1 amplification are 5′-AAATGGTGAAGGTCGGTGTGAA-3′ (forward) and 5′-CAACAATCTC CACTTTGCCACTG-3′ (reversed); primers used for Snap25 amplification are 5′-AGGACTTTGGTTATGTTGGAT-3′ (forward) and 5′-GATTTAAGCTTGTT ACAGG-3′ (reversed); primers used for Nox1 amplification are 5′-CTTTAGCATCCATATCCGCATT-3′ (forward) and 5′-GACTGGTGGCATTGTC ACAATA-3′ (reversed); primers used for Bcl2 amplification are 5′-ACGAGTGG GATACTGGAGATG-3′ (forward) and 5′-TAGCGACGAGAGAAGTCATCC-3′ (reversed); primers used for Kdr amplification are 5′-TCACGGTTGGGCTACTGC-3′ (forward) and 5′-AGACCTTCTGCCATCACG-3′ (reversed); primers used for Foxo3 amplification are 5′-TGGATGACCTGCTAGATAACAT-3′ (forward) and 5′-AACACGGTACTGTTAAAGGAGC-3′ (reversed); primers used for Notch3 amplification are 5′-GCTGGCGTCTCTTCAACAACA-3′ (forward) and 5′-TGGTCGGCGCAGTACTTCTTAT-3′ (reversed); primers used for *β*-actin (reference gene used in this study) amplification are 5′-AGGGAAATCG TGCGTGACAT-3′ (forward) and 5′-GAACCGCTCATTGCCGATAG-3′ (reversed).

### 2.8. Enrichment Analysis of DEGs

In order to understand the biological functions and pathways of DEGs in rat cerebral ischemia after PN intervention, we used an annotation and visualization integrated OmicShare cloud platform to analyze and visualize the biological functions and pathways of differential genes.

### 2.9. Statistical Analysis

All data are expressed as mean ± SEM, and statistical analysis was performed using the SPSS 22.0 statistical software (IBM, Chicago, Illinois). The experimental data were analyzed by one-way analysis of variance. Student's paired *t*-test was used to compare qPCR results, and *P* values < 0.05 were considered statistically significant. GO Analysis was based on the GO database. Fisher's exact test and multiple comparison test were used to calculate the significance level (*P* value) and false positive rate (FDR) of each function. *P* value < 0.05 was the criterion for significance screening. Pathway analysis was based on the KEGG database, and Fisher's exact test and chi-square test were used for the DEGs. A pathway in which the target gene participated in was analyzed for significance and selected according to *P* value < 0.05.

## 3. Results

### 3.1. The Effects of PN on MCAO in Rats Infarction Volume

**Figure 1:**
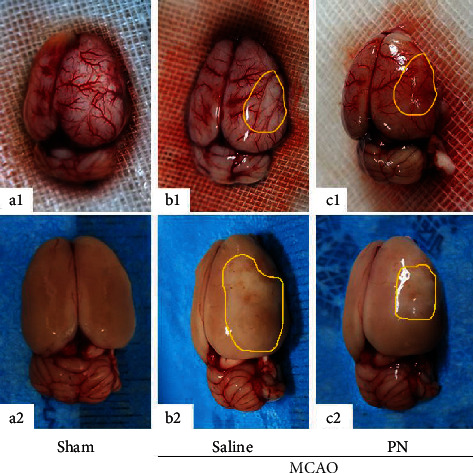
Brain tissue samples of rats from each group. (a) Sham group, (a1) cerebral vessels are abundant and clearly visible, (a2) brain tissue is full and symmetrical. (b) MCAO group treated with saline, (b1) blood vessels around the infarction show exudation, blocked blood vessels are seen to be collapsed and atrophied, and (b2) brain tissue is swollen and white. (c) MCAO group treated with PN, (c1) blood vessels around the infarction are mostly new, with less exudation than MCAO group and (c2) brain tissue is not swollen obviously. The yellow lines indicate the infarct area. Similar experiments were performed at least 5 times.

**Figure 2:**
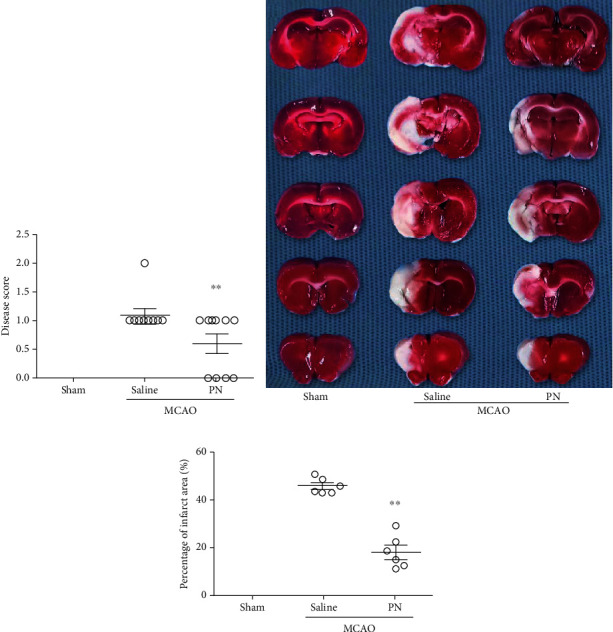
The effects of PN on MACO in rat infarction volume. (a) Compared with the MCAO group treated with saline, scores of neural function were decreased in the PN group (*n* = 10 in each group). (b) TTC staining, normal tissues are red and infarcted tissues are white. PN can effectively reduce the range of cerebral infarction in rats. (c) Compared with MCAO group, the infarct area and percentage in the PN group decreased significantly (*n* = 6 in each group). In (a) and (c), data shown indicated as mean ± SEM values. Similar experiments were performed at least 3 times. ^∗^*P* < 0.05; ^∗∗^*P* < 0.01; ^∗∗∗^*P* < 0.001.

In order to evaluate the protective effect of PN on cerebral ischemia, on the seventh day of the experiment, the neurological functions were assessed blindly using the Longa Neurological Severity Score; the brain was removed after anesthesia (Figure 1**)** and subjected to TTC staining in order to evaluate the scope of infarction (Figure 2). Comparing with the MCAO group treated with saline, PN-treated animals showed decreased disease score (Figure 2(a)) and significantly less infarct area (Figures 2(b) and 2(c)).

### 3.2. Ultrastructural Regulation of the NVU after PN Treatment

**Figure 3:**
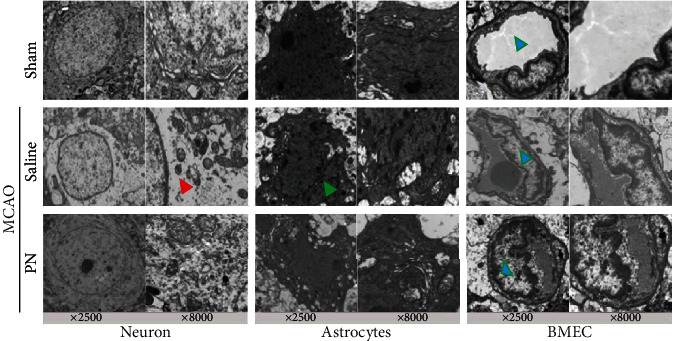
The effect of PN on ultrastructural changes of NVU. Similar experiments were performed at least 4 times. BMEC represents brain microvascular endothelial cell. The red triangle indicates a severe swollen/inflammation around neuron, the green triangle indicates an irregular nucleus. The blue triangle indicates the lumen side of brain microvascular.

Ultrastructural changes in the NVU were observed after 7 days of cerebral ischemia.  Figure 3 shows in the Sham group the neurons, and astrocytes are clearly visible and have large and rounded nuclei. The microvascular endothelial cells are clear structures without edema around them, and the lumen is normal. At high magnification, the binuclear membrane appears clear and complete with a clear field of view of the integrity of the cell structure. Organelles, such as lysosomes, are distributed throughout the cytoplasm. The surface of vascular endothelial cells is smooth and flat; and the endothelium, basement membrane, and foot processes are in close contact.

In the MCAO group, the neurons and astrocytes are irregular and show an accumulation of chromatin. Cytoplasm lysis and vacuole formation were observed. There appears to be estrogen receptor expansion, mitochondrial bending, disorder, shrinkage, and vacuolation in the cytoplasm. Mitochondrial and other organ-related injuries were more severe than those in the treatment group. Edema around blood vessels is obvious, showing vacuoles or blank areas (Figure 3).

In the PN group, the nuclei of neurons appear more normal with the nuclear membrane being intact, and the nucleoli are visible. The astrocyte structure is more complete than in the MCAO group with the nuclear morphology being more complete and clear, the microvascular endothelial cells are not deformed significantly, and the lumen is basically unobstructed. The peripheral edema is reduced compared with the MCAO group (Figure 3).

### 3.3. Gene Expression and Transcript Data Analysis

**Figure 4:**
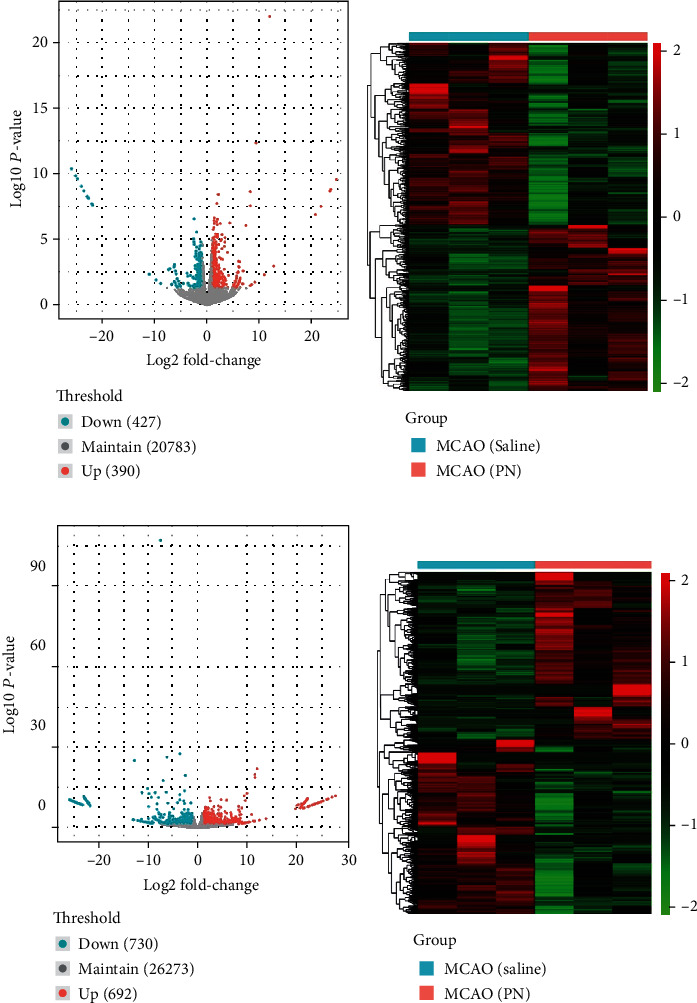
Volcano plots and hierarchical clustering heat map of DEGs and DETs. (a) Volcano map for DEGs where red represents upregulated genes and green represents downregulated genes. (b) Heat map for hierarchical clustering of DEGs. (c) Volcano map for DETs, red represents upregulated transcripts, and green represents downregulated transcripts. (d) Heat map for hierarchical clustering of DETs). *n* = 3 in each group.

To investigate the molecular mechanism of the protective effect of PN on MCAO in rats, brain tissue gene expression profiles of the Sham, MCAO, and PN groups were measured by using RNA-Seq. A total of 817 DEGs were observed in MCAO group compared to PN group, of which, 390 genes were upregulated and 427 were downregulated (Figures 4(a) and 4(b), Table 1). In addition, there were 1422 differentially expressed transcripts (DETs) which included 692 upregulated and 730 downregulated transcripts (Figures 4(c) and 4(d), Table 2).

### 3.4. RNA-Seq Validation by qPCR

**Figure 5:**
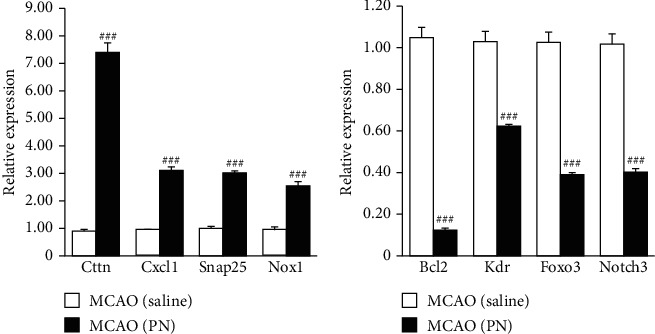
Verification of DEGs obtained from RNA-Seq by qPCR. Significance level according to nonparametric test (Cruskal-Wallis test, *n* = 6 in each group). Data shown indicated as mean ± SEM values. Similar results were obtained from at least 3 independent experiments. ^∗^*P* < 0.05; ^∗∗^*P* < 0.01; ^∗∗∗^*P* < 0.001.

To verify the expression of the DEGs obtained from the RNA-Seq results between MCAO and PN groups, we randomly selected 8 genes for validation, including 4 upregulated genes (Cttn, Cxcl1, Snap25, and Nox1) and 4 downregulated genes (Bcl2, Kdr, Foxo3 and Notch3). The relative expression was determined by the 2^−*△△*Ct^ method, and the results were consistent with the expression trends seen during RNA-Seq analysis (Figure 5). The qPCR results were consistent with the sequencing experiments.

### 3.5. GO and Pathway Analyses

In order to determine the functions and pathways of DEGs and DETs related to PN treatment, we performed GO and pathway analysis. Using *P* < 0.01 identified 332 GO terms, 202 of which were related to nerves and 55 were related to blood vessels, of which the following GO terms: cellular process (GO:0009987), biological regulation (GO:0065007), metabolic process (GO:0008152), regulation of biological process (GO:0050789), response to stimulus (GO:0050896), binding (GO:0005488), catalytic activity (GO:0003824), transcription regulator activity (GO:0140110), molecular transducer activity (GO:0060089), molecular function regulator (GO:0098772), cell (GO:0005623), cell part (GO:0044464), organelle (GO:0043226), membrane (GO:0016020), and organelle part (GO:0044422) were significantly different between saline and PN-treated groups. This indicates that the above biological processes are potentially related to the therapeutic function of PN.

**Figure 6:**
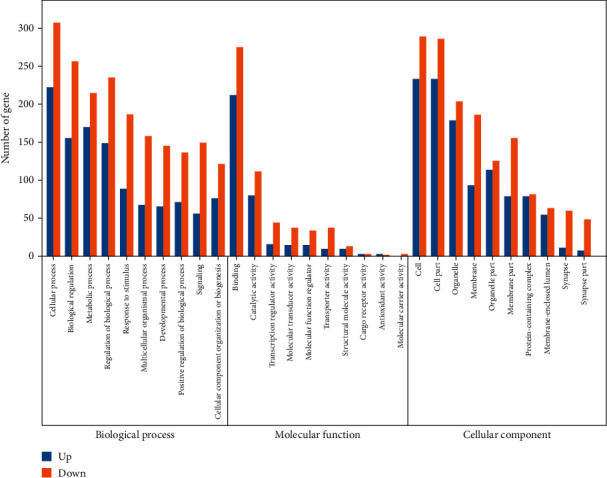
Gene ontology enrichment analysis of DEGs. The ordinate is the number of genes, the abscissa is the GO name. Blue refers to the upregulated genes; orange refers to the downregulated genes.

**Figure 7:**
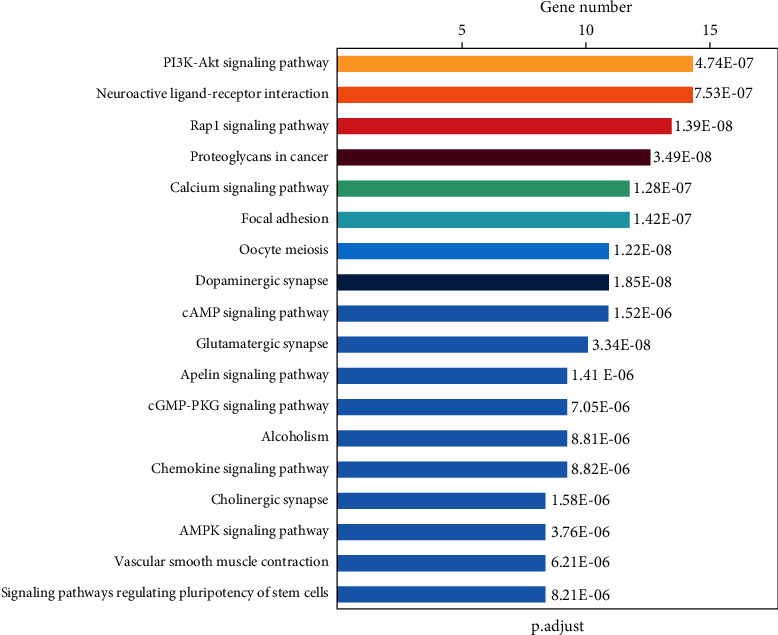
The list of top 18 enriched pathway analysis of DEGs.

**Figure 8:**
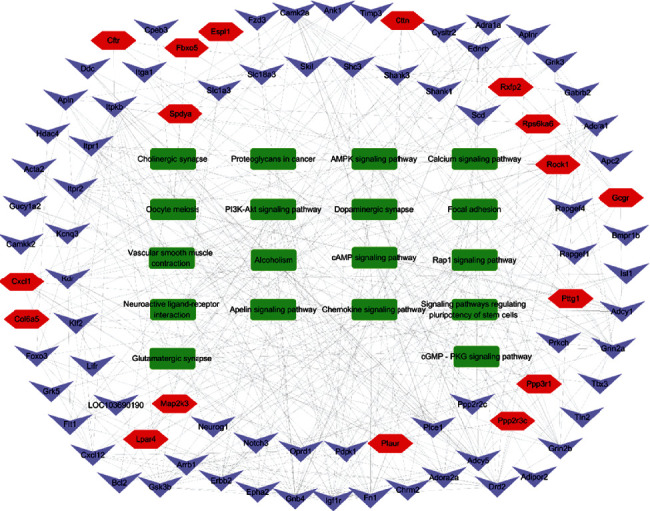
The important pathways and gene network map of PN anti-ischemic stroke. Green represents the pathways, purple represents the downregulated genes, and red represents the upregulated genes.

According to the enrichment degree of functional annotation, the top 10 biological processes, molecular functions, and cellular composition are shown in Figure 6 and Table 3. KEGG pathway analysis leads to a better understanding of the role of PN in the treatment of cerebral ischemia. 39 pathways were screened with *P* values of less than 0.05, of which 18 pathways were enriched with more than 10 genes. These include PI3K-Akt (ko04151), Rap1 (ko04015), neuroactive ligand-receptor interaction (ko04080), calcium (ko04020), focal adhesion (ko04510), oocyte meiosis (ko04114), cAMP (ko04024), glutamatergic synapse (ko04724), dopaminergic synapse (ko04728), apelin (ko04371), cGMP - PKG (ko04022), chemokine (ko04062), cholinergic synapse (ko04725), vascular smooth muscle contraction (ko04270), AMPK (ko04152), and signaling pathways regulating pluripotency of stem cells (ko04550) (Figure 7), and the genes participated in the above pathways were listed in Table 4. These pathways and genes may play an important role in the anti-ischemic effect of PN (Figure 8).

## 4. Discussion

It has been proven that PN possesses therapeutic properties against stroke, and it has been reported in previous studies about the molecular mechanism(s) explaining PN's clinical outcomes [11–13]. However, most of previous research focused on single a gene/pathway, still no systemic evaluation of PN's molecular mechanism(s) in neuroprotection effect. In this case, based on a rat stroke model treated with PN, we employed the second generation sequencing technology, which is a high-throughput method, in combination of qPCR and transmission electron microscopy; this study identified 817 DEGs potentially related to PN's neuroprotection effect, and we firstly identified 18 pathways involved in its therapeutic effect.

The development of stroke involves energy metabolism disorder, inflammatory response, free radical formation, calcium overload and apoptosis, and destruction of blood-brain barrier [2]. These adverse factors cause pathological damage to neurons, glial cells, and microvessels in the NVU [27].

In this study, we investigated the protective effect of PN on ischemic stroke in rats. Compared with the MCAO model group, PN treatment can significantly decrease the infarct volume/size in rats with cerebral ischemia and reduce the volume of cerebral infarction as shown by TTC staining. Hence, PN has an anti-ischemic effect. Ultrastructural results show that PN can reduce the pathological changes of the NVU in ischemic stroke.

KEGG pathway analysis showed PN's therapeutic effect involves various pathways such as PI3K-Akt pathway, Neuroactive ligand-receptor interaction, Rap1 signaling pathway, proteoglycans in cancer, calcium signaling pathway, focal adhesion, oocyte meiosis, cAMP signaling pathway, Glutamatergic synapse, Dopaminergic synapse, Apelin signaling pathway, cGMP-PKG signaling pathway, Alcoholism, Chemokine signaling pathway, Cholinergic synapse, Vascular smooth muscle contraction, and AMPK signaling pathway. Nine of which were firstly reported in this research to be related to PN.

It has been reported that PN exerts different molecular mechanisms and pathways related to its neuroprotective effect. Panax notoginseng saponins are the representative bioactive agent of PN extracts, and it is widely used in the treatment of ischemic stroke, probably due to its inhibition of apoptosis *via* upregulation of SIRT1 and antioxidants [28]. PN and its extract are known to have a variety of protective neurovascular mechanisms. For example, PNS protects cerebral microvascular endothelial cells by activating the PI3K/Akt/Nrf2 antioxidant signal pathway [29]. Notoginsenoside R1 plays a neuroprotective role by activating the estrogen receptor-dependent Akt/Nrf2 pathway to inhibit NADPH oxidase activity and mitochondrial dysfunction [30]. Ginsenoside Rb1 can upregulate the expression of GDNF to inhibit neuron apoptosis [31] and promote motor function recovery and axon regeneration in mice after stroke through the cAMP/PKA/CREB signaling pathway [32]. Ginsenoside Rd has been shown to inhibit microglial proteasome activity and inflammatory response [33, 34] through mitochondrial protection, apoptosis inhibition, and energy recovery. Ginsenoside Rg1 can regulate the inhibition of NMDA receptor channels and L-type voltage-dependent calcium channels on Ca^2+^ influx and the decrease of intracellular-free Ca^2+^ caused by it, thus playing a neuroprotective role [2]. Studies have also shown that the cGMP-PKG pathway mediates the proliferation of neural stem cells after cerebral ischemia [35]. Ras-associated protein 1 (Rap1) is known to be involved in integrin and cadherin-mediated adhesion, which can mediate angiogenesis in endothelial cells [36].

In our research, we identified another 9 pathways potentially related to PN's neuroprotective effect, including Neuroactive ligand-receptor interaction, proteoglycans in cancer, focal adhesion, oocyte meiosis, Apelin signaling pathway, Alcoholism, Chemokine signaling pathway, Cholinergic synapse, and Vascular smooth muscle contraction.

Genetically modified animals should be used to confirm the biological/pathological role of these newly identified pathways related to PN's therapeutic effect against stroke.

## 5. Conclusions

The NVU is the structural and functional unit of the nervous system. Our study shows that PN has a protective effect on cerebral ischemia by protecting the NVU. The molecular mechanisms involved may include the PI3K-Akt pathway, neuroactive ligand-receptor interactions, Rap1 signaling pathway, cAMP signaling pathway, and cGMP-PKG signaling pathway. The interaction of proteins in these pathways may be potential key targets for our future understanding of the neuroprotective effects of PN in ischemic stroke.

## Figures and Tables

**Table 1:** The partially DEGs from RNA-Seq [MCAO(Saline)/MCAO(PN)].

Ensembl gene ID	Gene symbol	Log2-fold change	*P* value	*P* adj	Style
ENSRNOG00000050994	Cttn	12.67967226	0.001172369	0.104286972	Up
ENSRNOG00000002802	Cxcl1	2.948761024	0.007257485	0.253659663	Up
ENSRNOG00000006037	Snap25	1.675988534	0.0003127	0.046747614	Up
ENSRNOG00000048706	Nox1	1.96651	0.011922266	0.31373928	Up
ENSRNOG00000002791	Bcl2	-1.777692036	0.003557504	0.176645472	Down
ENSRNOG00000046829	Kdr	-1.524693848	0.000278591	0.044899468	Down
ENSRNOG00000000299	Foxo3	-1.159297737	0.035026006	0.476124441	Down
ENSRNOG00000004346	Notch3	-1.450412508	0.014108102	0.337843688	Down

**Table 2:** The partially DETs from RNA-Seq [MCAO(Saline)/MCAO(PN)].

Transcript ID	Gene ID	Gene symbol	Log2-fold change	*P* value	*P* adj	Style
ENSRNOT00000000508	ENSRNOG00000000439	Ager	3.050138008	0.022180829	0.401239754	Up
ENSRNOT00000000553	ENSRNOG00000000473	Pfdn6	2.208199784	0.002663896	0.121543007	Up
ENSRNOT00000000657	ENSRNOG00000000546	Nt5dc1	1.080452666	0.036038318	0.50948505	Up
ENSRNOT00000000817	ENSRNOG00000000657	Nek7	3.035450677	0.001253867	0.070869068	Up
ENSRNOT00000002487	ENSRNOG00000001816	Rfc4	1.358324579	0.00530658	0.182793209	Up
ENSRNOT00000000205	ENSRNOG00000024631	Chadl	-1.127386831	0.006102632	0.202410056	Down
ENSRNOT00000000824	ENSRNOG00000000661	Hps4	-2.446591496	0.004995283	0.177592248	Down
ENSRNOT00000001185	ENSRNOG00000000886	Caln1	-1.460830778	0.015749777	0.338238335	Down
ENSRNOT00000001248	ENSRNOG00000000940	Flt1	-1.41782469	0.001002321	0.059826058	Down
ENSRNOT00000001479	ENSRNOG00000001117	Fbxl18	-1.477639058	0.020547561	0.386592868	Down

**Table 3:** GO classification of DEGs in MCAO(Saline)/MCAO(PN) (Top30).

Term	Description	Type	Count	*P* value
GO:0031323	Regulation of cellular metabolic process	BP	218	2.85E-06
GO:0001568	Blood vessel development	BP	40	2.99E-06
GO:0001944	Vasculature development	BP	41	3.22E-06
GO:0006836	Neurotransmitter transport	BP	23	3.64E-06
GO:0080090	Regulation of primary metabolic process	BP	213	3.85E-06
GO:0019222	Regulation of metabolic process	BP	230	3.92E-06
GO:0065008	Regulation of biological quality	BP	153	4.21E-06
GO:0072358	Cardiovascular system development	BP	41	4.92E-06
GO:0050905	Neuromuscular process	BP	14	5.87E-06
GO:0007626	Locomotory behavior	BP	20	6.12E-06
GO:0003676	Nucleic acid binding	MF	163	1.31E-06
GO:0097159	Organic cyclic compound binding	MF	233	1.29E-05
GO:1901363	Heterocyclic compound binding	MF	230	1.40E-05
GO:0046872	Metal ion binding	MF	138	1.89E-05
GO:0043169	Cation binding	MF	141	2.04E-05
GO:0005488	Binding	MF	488	5.61E-05
GO:0016263	Ion binding	MF	200	0.002322647
GO:0043565	Sequence-specific DNA binding	MF	53	0.001285424
GO:0003677	DNA binding	MF	85	0.001632778
GO:0003690	Double-stranded DNA binding	MF	44	0.001941181
GO:0045202	Synapse	CC	70	3.86E-08
GO:0044456	Synapse part	CC	55	2.90E-06
GO:0098794	Postsynapse	CC	40	4.36E-06
GO:0098984	Neuron to neuron synapse	CC	23	0.000185124
GO:0005622	Intracellular	CC	454	0.000264158
GO:0045211	Postsynaptic membrane	CC	20	0.000272495
GO:0097060	Synaptic membrane	CC	25	0.000310002
GO:0098978	Blutamatergic synapse	CC	26	0.000423116
GO:0032279	Asymmetric synapse	CC	21	0.000463989
GO:0034707	Chloride channel complex	CC	6	0.000580336

^∗^BP: biological process; MF: molecular function; CC: cellular component; Count: gene number listed in GO term.

**Table 4:** A list of the top 18 KEGG pathway DEGs.

Pathway ID	Pathway	Genes
ko04151	PI3K-Akt signaling pathway	Lpar4, Col6a5, Ppp2r3c, Foxo3, Flt1, Bcl2, Gsk3b, Pdpk1,Erbb2, Epha2, Gnb4, Igf1r, Fn1, Kdr, Chrm2, Itga1, Ppp2r2c.
ko04080	Neuroactive ligand-receptor interaction	Rxfp2, Lpar4, Gcgr, Adora2a, Adora1, Apln, Gabrb2, Drd2, Grin2b, Grik3, Aplnr, Adra1a, Oprd1, Ednrb, Cysltr2, Grin2a, Chrm2.
ko04015	Rap1 signaling pathway	Lpar4, Map2k3, Flt1, Adora2a, Rapgef4, Adcy5, Drd2, Grin2b, Epha2, Igf1r, Plce1, Rapgef1, Tln2, Grin2a, Kdr, Adcy1.
ko05205	Proteoglycans in cancer	Rock1, Plaur, Cttn, Itpr2, Timp3, Pdpk1, Erbb2, Itpr1, Igf1r, Plce1, Fn1, Ank1, Camk2a, Kdr, Fzd3.
ko04020	Calcium signaling pathway	Ppp3r1, Adora2a, Itpr2, Itpkb, Erbb2, Itpr1, Adra1a,Ednrb, Plce1, Cysltr2, Camk2a, Grin2a, Chrm2,A dcy1.
ko04510	Focal adhesion	Col6a5, Rock1, Flt1, Bcl2, Gsk3b, Pdpk1, Erbb2, Igf1r, Fn1, Rapgef1, Shc3, Tln2, Kdr, Itga1.
ko04114	Oocyte meiosis	Rps6ka6, Pttg1, Spdya, Espl1, Fbxo5, Ppp3r1, Itpr2,Adcy5, Itpr1, Igf1r, Camk2a, Cpeb3, Adcy1.
ko04024	cAMP signaling pathway	Rock1, Cftr, Adora2a, Rapgef4, Adcy5, Adora1, Drd2, Grin2b, Plce1, Camk2a, Grin2a, Chrm2, Adcy1.
ko04724	Glutamatergic synapse	Ppp3r1, Arrb1,Itpr2,Adcy5, Itpr1, Grin2b, Grik3, Slc1a3, Shank1, Grin2a, Shank3, Gnb4, Adcy1.
ko04728	Dopaminergic synapse	Ppp2r3c, Itpr2, Adcy5, Gsk3b, Ddc, Itpr1, Drd2, Grin2b, Gnb4, Camk2a, Grin2a, Ppp2r2c.
ko04371	Apelin signaling pathway	Itpr2, Adcy5, Apln, Notch3, Itpr1, Aplnr, Gnb4, Klf2, Hdac4, Acta2, Adcy1.
ko04022	cGMP-PKG signaling pathway	Rock1, Ppp3r1, Itpr2, Adcy5, Adora1, Itpr1, Adra1a, Oprd1, Ednrb, Gucy1a2, Adcy1.
ko05034	Alcoholism	Adora2a, Camkk2, Adcy5, Ddc, Drd2, Grin2b, Gnb4, Shc3, Hdac4, Grin2a, LOC103690190.
ko04062	Chemokine signaling pathway	Cxcl1, Rock1, Foxo3, Adcy5, Gsk3b, Gnb4, Grk5, Cxcl12, Shc3, Arrb1, Adcy1.
ko04725	Cholinergic synapse	Itpr2, Adcy5, Bcl2, Kcnq3, Itpr1, Gnb4, Camk2a, Chrm2, Adcy1, Slc18a3.
ko04270	Vascular smooth muscle contraction	Rock1, Adora2a, Itpr2, Adcy5, Prkch, Itpr1, Adra1a, Gucy1a2, Acta2, Adcy1.
ko04152	AMPK signaling pathway	Ppp2r3c, Cftr, Foxo3, Camkk2, Pdpk1, Adipor2, Adra1a, Scd, Igf1r, Ppp2r2c.
ko04550	Signaling pathways regulating pluripotency of stem cells	Gsk3b, Tbx3, Skil, Lifr, Isl1, Igf1r, Bmpr1, Neurog1, Apc2, Fzd3.

## Data Availability

The datasets and code generated or analyzed in this study are available from the corresponding author upon reasonable request.

## Supplementary Materials

Supplementary Materials Supplementary Table: Longa's method of evaluation of neurological defects. The severity of neurological defects could be indicated by rat behaviors and was scored from 0 to 4.
