# Supplementary material for: Genes Induced by Panax Notoginseng in a Rodent Model of Ischemia-Reperfusion Injury
Source: J Immunol Res. 2020 Nov 25;2020:8873261. doi: 10.1155/2020/8873261 (PMC7714582; doi:10.1155/2020/8873261)
Supplement: Supplementary Materials — Supplementary Table: Longa's method of evaluation of neurological defects. The severity of neurological defects could be indicated by rat behaviors and was scored from 0 to 4. [file 8873261.f1.docx]

**Supplementary Table**

**Longa’s Method of Evaluation of Neurological Defects**

| **Rat Behavior** | **Neurological Defects** | **Disease Score** |
| --- | --- | --- |
| Normal | No neurological behavioral impairment | 0 |
| Right front paw couldn’t be fully extended | Mild functional impairment | 1 |
| Walking towards left (paralyzed) | Moderate impairment | 2 |
| Dumping to the left | Severe nerve damage | 3 |
| Couldn’t walk on itself | Loss of consciousness | 4 |
